# Sensitisation to *Imbrasia belina* (mopane worm) and other local allergens in rural Gwanda district of Zimbabwe

**DOI:** 10.1186/s13223-022-00668-0

**Published:** 2022-04-09

**Authors:** Vuyelwa Ndlovu, Moses Chimbari, Pisirai Ndarukwa, Elopy Sibanda

**Affiliations:** 1 School of Nursing and Public Health, College of Health Sciences, Howard College Campus, University of KwaZulu-Natal, Durban, South Africa; 2 Department of Environmental Science and Health, Faculty of Applied Sciences, National University of Science and Technology, Corner Gwanda Road and Cecil Avenue, PO Box AC 939, Ascot, Bulawayo Zimbabwe; 3 Department of Health Sciences and Faculty of Sciences, Bindura University of Science Education, P Bag 1020, Bindura, Zimbabwe; 4 Asthma, Allergy and Immune Dysfunction Clinic, Twin Palms Medical Centre, 113 Kwame Nkrumah Avenue, Harare, Zimbabwe; 5 Department of Pathology, Medical School, National University of Science and Technology, Bulawayo, Zimbabwe; 6 Great Zimbabwe University , P.O Box 1235, Masvingo, Zimbabwe

**Keywords:** Allergic sensitisation, Entomophagy, Mopane worm, Zimbabwe

## Abstract

**Background:**

The prevalence of allergic diseases is increasing in Zimbabwe and the data relate to local as well as exotic allergen sources. As entomophagy, the practice of eating insects, is a recognised source of local allergens, we sought to measure the prevalence of and risk factors for sensitisation to *Imbrasia belina* (mopane worm), a popular edible insect. This was investigated alongside other locally relevant allergens in a rural community in Gwanda district, south of Zimbabwe.

**Methods:**

A cross sectional study was conducted among 496 adults and children aged 10 years and above in Gwanda district, a mopane worm harvesting area in Zimbabwe. Data on individual characteristics and mopane worm exposure factors were collected using questionnaires. Sensitivity to allergens was assessed by performing skin prick tests at a local clinic using 10 different commercial allergen extracts (Stallergenes, France) and in-house extracts of mopane worm *(Imbrasia belina)* and mopane leaves (*Colophospermum mopane*). Data were analysed using Stata version 13 software.

**Results:**

The prevalence of sensitisation to at least one allergen was 31.17% (n = 144). The prevalence of atopy was higher in adults (33.33%) than in children (23.53%) (p = 0.059). The commonest inhalant allergen sources were mopane worm (14.29%), *Tyrophagus putrescentiae* (14.29%), mopane leaves (13.42%), *Alternaria alternata* (6.49%) and *Dermatophagoides pteronyssinus* (6.49%). Polysensitisation was demonstrated in the study population and of the 108 participants (75%) who were sensitised to two or more allergens, 66 (61%) were women. Sensitisation to mopane worm and mopane leaves often clustered with *Tyrophagus putrescentiae* amongst adults. Adjusted logistic regression analyses between mopane worm sensitisation and self-reported exposure variables showed that sensitisation was more likely amongst mopane worm harvesters (OR = 1.92, 95%CI = 0.77–4.79), those who cooked or roasted mopane worms during harvesting (OR = 2.69, 95%CI = 0.78–9.31) and harvesting without personal protective equipment (PPE) (OR = 2.12, 95%CI = 0.83–5.44) compared to non-harvesters.

**Conclusion:**

Atopic sensitization was common in this mopane worm harvesting community in Gwanda district of Zimbabwe. There was frequent co-sensitisation of mopane worm and mopane leaves with *Tyrophagus putrescentiae* in children and adults. It is important to determine the clinical relevance of our findings, particularly relating to mopane worm sensitisation.

**Supplementary Information:**

The online version contains supplementary material available at 10.1186/s13223-022-00668-0.

## Background

There is a rapid increase of allergic diseases occurring in African countries that if not addressed, could significantly increase the continent’s disease burden in the future [1, 2]. The increase in allergic diseases is attributed to climate, lifestyle and dietary changes that are occurring partly because of economic growth and development [3]. The pathogenesis of allergic diseases involves prior exposure and sensitisation to allergens that are sourced from the local environment [4–6]. Allergic sensitization or atopy, is an important risk factor for allergic disease. Atopy is defined as the genetic predisposition to produce immunoglobulin E (IgE) in response to exposure to common allergens [7]. In view of this, research is underway focusing on identifying clinically relevant environmental allergens and sensitisation patterns across a variety of populations and geographic locations. The findings are crucial for implementing evidence based prevention and control strategies for allergic diseases such as the design of informed awareness campaigns, allergen avoidance and the effective use of allergen specific immunotherapy [7]. Of concern, is the paucity of such studies in many African countries despite the increasing burden of allergic diseases [7].

In Zimbabwe only a few studies on allergic diseases have been conducted over the past four decades [8]. Although there are reports indicating an increasing prevalence of allergy in Zimbabwe [9–11], these studies suffer from a pro-urban and potentially an economic bias being derived from patients who visited hospitals or private clinics with suspected allergic symptoms. In Africa, very few studies have been done in rural areas [12, 13]. While it is believed that allergy prevalence is lower in rural areas due to lifestyle and environment factors in low and high income countries, the situation could be changing [3, 14].

It is against this background that we studied allergen sensitisation due to entomophagy and entomophagy related activities such as harvesting and storage of the insects [15]. The practice of harvesting *Imbrasia belina* or *Gonimbrasia belina*, a popular indigenous edible insect also known as ‘mopane worm,’ is common place in southern rural Zimbabwean communities. Locally, they are called ‘amacimbi’ or ‘madora’ in the Ndebele and Shona languages respectively. *Imbrasia belina* is an important source of food and economic livelihood for the rural communities [16]. Taxonomically, mopane worm is in the caterpillar phase of the emperor moth and that belongs to the *Saturniidae* family of the order *Lepidoptera* in the *Insecta* class [17, 18]. The name ‘mopane worm’ stems from the fact that the caterpillar feeds primarily, though not exclusively, on the leaves of the mopane tree (*Colophospermum mopane*) that is widely distributed throughout Southern Africa [19–24].

There has been increasing demand for this insect as entomophagy continues to grow in Zimbabwe [16, 25, 26]. Mopane worms are a lucrative business worth an estimated US$85 million annually in Southern Africa, contributing up to 25% of total annual cash income for rural households [24, 27]. The majority of edible insects in Africa are harvested through traditional means, a process also termed “wild harvesting” [28, 29]. In Zimbabwe, there are normally two harvests per year with the main harvest occurring in November–January and a smaller second harvest in April–May depending on the quantity of rainfall that year [16]. The harvesting process typically involves hand picking mopane worms from the Mopane tree branches followed by degutting, boiling or roasting and sun drying them [27]. While this has traditionally been an occupation for women and children, men are participating due to the increased commercial value of mopane worm. Surveys in Zimbabwe and Botswana reported that even young children less than 12 years old participate in mopane worm harvesting in various ways [30]. Children less than 10 years old, however, have less productive roles such as looking after babies at the campsite while the mothers harvest and process mopane worm [30].

Mopane worm harvesting has increased as a consequence of its increasing commercial value and the decreasing agricultural yields due to climate change [31]. The shift in harvesting practices to meet commercial demand raises health concerns because entomophagy is a recognised source of neo-allergens [32–35]. *Imbrasia belina* associated allergy has been documented in Botswana and Zimbabwe [36–38]. However, no studies have been carried out to investigate mopane worm sensitisation at population level particularly in vulnerable communities where exposure is highest.

The aim of this study was to determine the prevalence of and risk factors for sensitisation to mopane worm and other local environmental allergens among adults and children in a rural community in Gwanda district located in the southern region of Zimbabwe. We also analysed the co-sensitisation patterns emerging from allergens tested for.

## Materials and methods

### Study design and setting

This cross sectional study was conducted from October 2019 to March 2020 as part of the Gwanda Asthma and Respiratory Allergy Study (GARAS) that was designed to investigate the presence of asthma and respiratory allergy in Gwanda rural community and identify the associated demographic, environmental and socio-cultural factors. All procedures and tools were pre-tested in a feasibility study carried out prior to the main study [39]. The study was approved by the Medical Research Council of Zimbabwe (Reference number MRCZ/A/2486) and the University of KwaZulu-Natal’s Biomedical Research Ethics Committee (BREC) (Reference number BE 327/19).

### Study population and sampling

Based on local observations and previous surveys indicating that children as young as 10 years old participated in mopane worm harvesting, this study included participants aged 10 years and older residing in the study area, Garanyemba, Gwanda district for at least a year [30]. A non-probability, volunteer sampling strategy was used to recruit eligible participants with the assistance of the village leaders from each of the eight villages in Garanyemba. This approach was informed by a low response rate in the feasibility study [39]. Based on the assumption of a mopane worm sensitization estimate of 50%, a 5% margin of error at 95% confidence interval and an additional 20% to compensate for anticipated non-response, a sample size of 462 participants was calculated [40].

### Questionnaire

All data was collected in a centrally located clinic in Garanyemba [39]. Informed consent and, in cases of children, parental consent and the child’s assent were obtained from participants presenting at the clinic. Thereafter, questionnaires were administered to consenting participants by trained interviewers in the local isiNdebele language using Kobo Collect software [41]. The questionnaire addressed family history of allergy (history of allergy among parents and siblings); demographic and socio-economic characteristics (age, gender, level of education, monthly income); self-reported history of allergy (hay fever, eczema, asthma, allergy symptoms upon exposure to plants, furry animals); lifestyle factors (smoking and alcohol consumption) and mopane worm exposure factors.

### Mopane worm exposure assessment

Published literature detailing the mopane worm harvesting and post harvesting processes was used to identify possible routes of exposure and to develop appropriate questions for qualitative exposure assessment [16, 24, 27, 30]. These included self-report of harvesting activities such as picking, degutting, cooking/roasting and post harvesting activities included eating, storage and selling. The questionnaire also included questions on frequency (number of days per week of harvesting), duration (years of harvesting) of exposure to mopane worm and use of personal protective equipment (PPE) during harvesting. PPE during harvesting included the use of at least one of the following: apron, boots, gloves, mask, overalls and goggles.

### Assessment of sensitisation to inhalant allergens

Commercial inhalant allergen extracts (Stallergenes, France) considered for this study were House Dust Mites (*Dermatophagoides farina* and *Dermatophagoides pteronyssinus*), storage mite *(Tyrophagus putrescentiae*), Tree mixture (Maple, Horse chestnut, Plane, False acacia, Lime), Five Grass mix (cocksfoot, meadow, rye, sweet vernal, timothy), Weed mixture, Barley grass, Cockroach, Mosquito and *Alternaria alternata*. This panel of allergens was found to be locally relevant based on a previous study in Zimbabwe [9]. Mopane worm *(Imbrasia belina)* and mopane tree leaf extracts were prepared in-house using previously detailed procedures [39]. An extract of mopane tree leaves was included in the panel because mopane worms feed primarily, though not exclusively, on the leaves of the mopane tree, *Colophospermum mopane* [19]. For mopane worms, the extract was diluted using 0.9% sodium chloride to a concentration of 1.437 mg/dl and for Mopane leaves, the extract was diluted using 0.9% sodium chloride to a concentration of 0.5085 mg/dl. Protein concentration was determined using spectrophotometry. Histamine (10 mg/mL) and saline (0.9% NaCl) were also included in the panel as positive and negative controls, respectively.

Skin prick testing was performed by trained staff at the clinic as previously described [39]. Skin-prick test wheal diameters exceeding 3 mm or greater than the saline control was considered positive [42]. A participant was classified as atopic if sensitised to at least one allergen.

### Data analysis

We analysed the data using Stata Release 13 (StataCorp, Texas, USA) [43]. Variables were summarised using descriptive statistics. Analyses were stratified by age and gender, where appropriate, because of the anticipated systematic differences in environmental exposure patterns and susceptibility [44]. Mean and standard deviation or median and interquartile ranges were used to summarize the continuous variables that were normally or not normally distributed respectively. Categorical variables were summarised as frequencies and percentages. To compare prevalence of allergen sensitisation between the different age and gender groups, the chi-square test was used and in the event of an expected frequency of less than 5, the Fisher’s exact test was used. To compare mopane worm exposure variables and other covariates between adults and children, the chi-square test or the Fisher’s exact test for categorical variables. For numerical variables, the two sample t-test or Wilcoxon rank-sum test was used. Significance was considered for p values less than 0.05 for all the statistical tests performed.

The variable ‘Polysensitisation’ was the sum of allergens each individual was sensitised to ranging from a minimum of 0 to a possible maximum of 12. To statistically identify the patterns of co-sensitisation, we applied cluster detection techniques in the group of participants classified as atopic. Agglomerative hierarchical clustering was performed in Stata 13 using Ward’s linkage with the simple matching binary similarity coefficient to produce a dendrogram (Additional file 1: Fig. S1). The optimum number of clusters were selected from a visual assessment of the dendogram and application of the Caliński and Harabasz pseudo-F index stopping rule [45]. The clusters were then evaluated and profiled on a number of characteristics including age, gender and specific allergen sensitization (Additional file 1).

Multivariate logistic regression was used to estimate the odds ratios (ORs) and their 95% confidence intervals (CIs) to determine the association between mopane worm exposure variables and sensitization to mopane worm. The change-in-estimate (CIE) criterion was used to identify confounders that were defined as covariates that change the regression coefficient of the crude exposure-outcome relationship by at least 10% [46]. The covariates selected for inclusion in the final models were age, gender, education, polysensitisation and family history of allergy. Mopane worm exposure variables were then added separately to the mopane worm sensitisation outcome model adjusting for these covariates. A categorical variable was generated from duration of mopane worm harvesting whereby a period of 2 years was considered a reasonable period in which a neo-sensitisation can occur. This was derived from studies reporting sensitisation to neo-allergens following immunotherapy [47, 48]. Participants reporting a harvesting duration of less than 2 years, including no harvesting at all, were included in the comparison group for this variable. The variable on storage and selling of mopane worms was highly correlated with the variable on harvesting and the former was therefore dropped from the analysis to reduce multicollinearity. Variables with p < 0.05 in the final model were regarded as significantly associated factors.

## Results

A summary of demographic characteristics of study participants is presented in Table 1. The majority of participants (78%) were adults (aged ≥ 18 years), 22% were children (aged < 18 years). There were 347 (69.96%) females and 149 (30.04%) males with a roughly similar ratio of males to females (30:70) in both adults and children. The mean age in adults was 42.84 (± 18.06) years and in children it was 14.09 (± 2.03) years.

**Table 1:** Individual characteristics of the study population in rural Gwanda, Zimbabwe (n = 496)

Characteristic	Adults (n = 388)	Children (n = 108)	Total
*Demographic and socioeconomic characteristics*			
Participation by village, n (%)			
Garanyemba	104 (26.8)	30 (28.04)	134 (27.07)
Mawane 1	26 (6.7)	4 (3.74)	30 (6.06)
Mawane 2	33 (8.51)	4 (3.74)	37 (7.47)
Mtandawenhema	33 (8.51)	7 (6.54)	40 (8.08)
Nsimbi	77 (19.85)	15 (14.02)	92 (18.59)
Sifanjani	20 (5.15)	5 (4.67)	25 (5.05)
Swisha	50 (12.89)	26 (24.3)	76 (15.35)
Zhokwe	45 (11.60)	16 (14.95)	61 (12.32)
Total	388 (78.23)	108 (21.77)	496 (100)
Age, mean(sd)	42.84 (18.06)	14.09 (2.03)	36.58 (19.92)
Gender, n (%)			
Male	115 (29.64)	34 (31.48)	149 (30.04)
Female	273 (70.36)	74 (68.52)	347 (69.96)
Education (n = 490), n (%)^a^			
No education	23 (6)	0 (0)	23 (4.69)
Primary	147 (38.48)	34 (31.48)	181 (36.94)
Secondary and tertiary	212 (55.50)	74 (68.52)	286 (58.37)
Monthly household income below poverty line (n = 354), n (%)^a,b^	354 (100)	–	–
Ever smoked	51 (13.35)	1 (0.95)	52 (10.68)
Passive smoking	196 (51.31)	36 (33.96)	232 (47.54)
Alcohol use	60 (15.79)	0 (0)	60 (12.3)
*Individual and family history of allergy*			
Allergic sensitization (n = 462), n (%)^**c**^	120 (33.33)	24 (23.53)	144 (31.17)
Any allergy, n (%)	129 (33.77)	29 (26.85)	158 (32.24)
Nasal allergy, n (%)	107 (28.01)	23 (21.3)	130 (26.53)
Skin allergy, n (%)	59 (15.49)	11 (10.19)	70 (14.31)
Allergy symptoms when harvesting mopane worms (n = 309), n (%)	70 (26.82)	12 (25)	82 (26.54)
Allergy symptoms near grass, flowers or trees n (%)	131 (34.29)	23 (21.50)	154 (31.49)^*^
Allergy symptoms near furry animals, n (%)	155 (40.58)	35 (32.71)	190 (38.85)
History of allergy among siblings, n (%)	124 (32.63)	20 (18.52)	144 (29.51)^*^
History of allergy among parents, n (%)	85 (22.31)	13 (12.04)	98 (20.04)^*^

^a^There were missing data (n < 496) in some variables

^b^The World Bank’s International Poverty Line of US$1.90/person/day was used and children were not asked about monthly household cash income

^c^(p = 0.059) and 462 participants consented to SPTs

*p < 0.05

All children were either in primary or secondary school whereas amongst adults, there was a small group (6%) that had never been to school. Passive smoking was common with a total of 232 (47.54%) participants reporting exposure. The individual and family history of allergy was also presented in Table 1. The prevalence of any allergy (hay fever, itchy and watery eyes/nose, eczema or any kind of skin allergy) was 32.24%. The prevalence of self-reported symptoms suggestive of hay fever was higher (26.53%) than that of skin related allergies (14.31%). Self-reported allergy symptoms upon exposure to plants or furry animals and family history of allergy was generally higher in adults than in children.

Of the total of 496 participants who completed the questionnaires, 462 underwent skin prick tests, giving a response rate of 93.15%. The overall prevalence of sensitization was 31.17%. The prevalence of sensitization was higher in adults (33.33%) than in children (23.53%) (p = 0.059). Table 2 summarises the prevalence of allergen specific sensitisation among adults and children. The most common sensitising inhalant allergen sources were mopane worm and *Tyrophagus putrescentiae* with prevalence of 14.29% each, followed by mopane leaves (13.42%), *Alternaria alternata* and *Dermatophagoides pteronyssinus* had a prevalence of 6.49%, each. The least common allergen source was the five grass mix (2.81%).

**Table 2:** Comparison of the prevalence of allergen specific sensitisation among adults and children in the study

Allergens, n (%)	Adults (n = 360)			Children (n = 102)			Total (n = 462)
	Female (n = 251)	Male (n = 109)	Total	Female (n = 71)	Male (n = 31)	Total	
Barley	17 (6.77)	7 (6.42)	24 (6.67)	2 (2.82)	0 (0)	2 (1.96)	26 (5.63)
5 grass mixture	7 (2.79)	4 (3.67)	11 (3.06)	2 (2.81)	0 (0)	2 (1.96)	13 (2.81)
Tree mixture	12 (4.78)	9 (8.26)	21 (5.83)	4 (5.63)	1 (3.23)	5 (4.90)	26 (5.63)
Cockroach (B. germanica)	17 (6.77)	6 (5.5)	23 (6.39)	2 (2.82)	0 (0)	2 (1.96)	25 (5.41)
Mosquito	17 (6.77)	4 (3.67)	21 (5.83)	1 (1.41)	0 (0)	1 (0.98)	22 (4.76)
HDM (D.farinae)	17 (6.77)	4 (3.67)	21 (5.83)	2 (2.82)	0 (0)	2 (1.96)	23 (4.98)
HDM (D.pter)	20 (7.97)	7 (6.42)	27 (7.5)	3 (4.23)	0 (0)	3 (2.94)	30 (6.49)
Tyrophagus putrescens*	40 (15.94)	22 (20.18)	62 (17.22)	4 (5.63)	0 (0)	4 (3.92)	66 (14.29)
Alternaria alternata	17 (6.77)	9 (8.26)	26 (7.22)	4 (5.63)	0 (0)	4 (3.92)	30 (6.49)
Weed mixture	9 (3.59)	7 (6.42)	16 (4.44)	3 (4.23)	0 (0)	3 (2.94)	19 (4.11)
Mopani worm	36 (14.34)	18 (16.51)	54 (15)	10 (14.08)	2 (6.45)	12 (11.76)	66 (14.29)
Mopane tree leaves	33 (13.15)	16 (14.68)	49 (13.61)	10 (14.08)	3 (9.68)	13 (12.75)	62 (13.42)

*p < 0.05

While prevalence of sensitisation was higher among adults compared to children for all the specific allergens studied, it was only with *Tyrophagus putrescentiae* that the difference was statistically significant (p < 0.05). Among adults, *Tyrophagus putrescentiae* was the commonest sensitiser (17.22%), followed by mopane worm (15%) and mopane leaves (13.61%). Children were mostly sensitised to mopane leaves (12.75%) and mopane worm (11.76%). Boys were generally the least sensitised population group in this study while girls contributed the most towards overall burden among children.

**Fig. 1:**
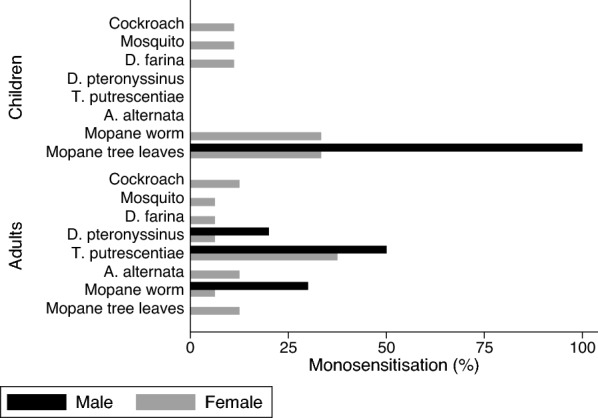
The distribution of monosensitisation among adults and children. This bar chart excludes Tree mixture (Maple, Horse chestnut, Plane, False acacia, Lime), Five Grass mixture (cocksfoot, meadow, rye, sweet vernal, timothy), Weed mixture and barley grass as none of the participants were monosensitised to those allergens

There were age and gender differences in the patterns of sensitisation observed in the sensitized group (n = 144). Sensitization to only one allergen (monosensitisation) was present in 36 (25%) participants composed of adult females 16 (44%), males 10(28%), girls 9 (25%), and boys 1 (3%). Out of the 36 mono-sensitised participants, 26 (72%) were adults and they were predominantly sensitised to *Tyrophagus putrescentiae* (42.31%), mopane worms (15.38%) and *D. pteronyssinus* (11.54%). Children were mostly sensitised to mopane worms (30%) and mopane leaves (40%) as shown in Fig. 1.

**Fig. 2:**
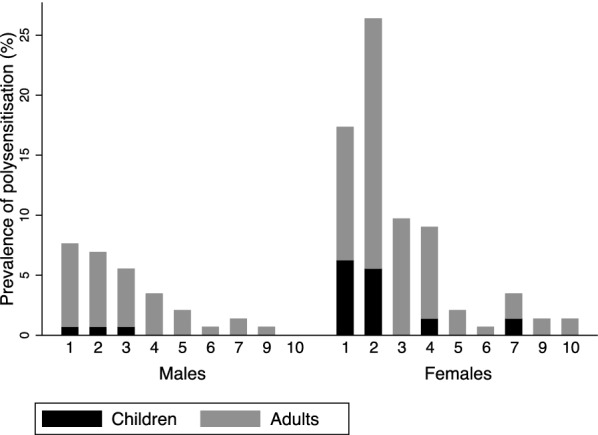
Sensitisation patterns indicating the distribution of polysensitisation to allergens (stratified by gender). Each bar represents total prevalence of those sensitised to the specified number of allergens and simultaneously showing the distribution of that total between adults and children

**Fig. 3:**
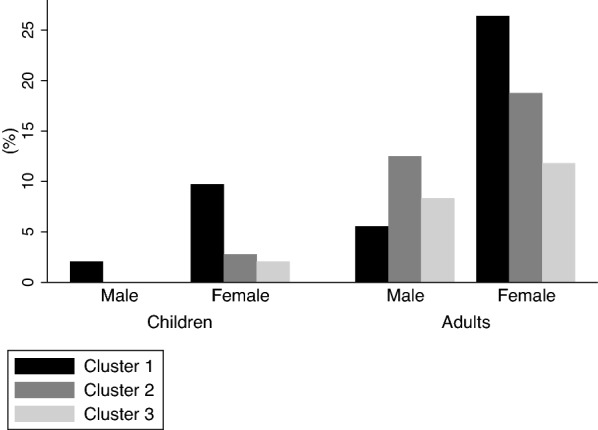
Frequency distribution of the 3 clusters. Stratification was by age and gender. In cluster 1, the dominant allergens are mopane worm and mopane leaves. In cluster 2, the dominant allergens were *Tyrophagus putrescentiae* and *Alternaria alternata* and in cluster 3, the dominant allergens were mopane worm, mopane leaves, *Tyrophagus putrescentiae* and Trees mixture

Figure 2 shows the distribution, stratified by age and gender according to the number of allergens to which participants were sensitized ranging from 1 to 10 for females and 1 to 9 for males. Polysensitisation was most common amongst those sensitised to 2, 3 and 4 allergens with prevalence of 48(33%), 22(15.28%) and 18(12.5%) respectively (Fig. 2). Furthermore, among the 108 participants sensitised to at least 2 allergens, women 66(61%) were the most polysensitised group followed by men 28(26%), girls 12(11%) and boys 2(2%). The cluster analysis of 144 atopic participants, revealed 3 sensitization clusters. A detailed profile of the clusters is presented in Additional file 1. The participants in cluster 1 and cluster 2 were predominantly sensitised to 2 allergens whereas cluster 3 was characterised by higher levels of polysensitisation with a median number of 4 allergens (Additional file 1: Table S1). The dominant allergens in cluster 1 were mopane worm and mopane leaves. In cluster 2, the dominant allergens were *Tyrophagus putrescentiae* and *Alternaria alternata*. In cluster 3, the four most frequent allergens were mopane worm, mopane leaves, *Tyrophagus putrescentiae* and trees mixture. The gender and age distribution of these 3 clusters is presented in Fig. 3. Most of the children in this study belonged to cluster 1 (mopane worm/mopane leaf sensitized) and there were more females (both girls and women) than males in cluster 1 compared to the other two clusters. Self-reported mopane worm exposure factors were stratified by age and gender as shown in Table 3.

**Table 3:** Mopane worm exposure variables among adults and children in the study

Variables	Adults, n (%)			Children, n (%)			Total population, n (%)
	Female (n = 251)	Male (n = 109)	Total (n = 388)	Female (n = 71)	Male (n = 31)	Total (n = 108)	Total (n = 462)
Eat mopane worm	185 (67.77)	83 (72.17)	268 (69.07)	51 (68.92)	32 (94.12)*	83 (76.85)	351 (70.77)
Harvest mopane worm*	185 (67.77)	77 (66.96)	262 (67.53)	26 (35.14)	22 (64.71)*	48 (44.44)	310 (62.50)
*Harvesting activities among harvesters*	*(n = 185)*	*(n = 77)*	*(n = 262)*	*(n = 26)*	*(n = 22)*	*(n = 48)*	*(n = 310)*
Picking mopane worm	114 (61.62)	50 (64.94)	164 (62.60)	16 (61.54)	16 (72.73)	32 (66.67)	196 (63.23)
Degutting mopane worm	27 (14.59)	7 (9.09)	34 (12.98)	5 (19.23)	4 (18.18)	9 (18.75)	43 (13.87)
Cooking mopane worm	44 (23.78)	20 (25.97)	64 (24.43)	5 (19.23)	2 (9.09)	7 (14.58)	71 (22.90)
PPE during harvesting	30 (16.22)	10 (12.99)	40 (15.27)	5 (19.23)	6 (27.27)	11 (22.92)	51 (16.45)
Harvesting/week*							
1 day	2 (1.08)	3 (3.95)	5 (1.92)	1 (3.85)	3 (13.64)	4 (8.33)	9 (2.91)
2–3 days	40 (21.62)	24 (31.58)	64 (24.52)	17 (65.38)	13 (59.09)	30 (62.5)	94 (30.42)
4–7 days	143 (77.30)	49 (64.47)^a^	192 (73.56)	8 (30.77)	6 (27.27)	14 (29.17)	206 (66.67)
Years of harvesting, mean (sd)*	7 (3–15)	5 (2–12)	6 (3–15)	2 (1–2)	2 (1–3)	2 (1–2.5)	5 (2–12)

*p < 0.05; ^a^p = 0.058

Out of the 496 participants, 351(70.77%) reported that they ate mopane worms. Among children, a significantly higher proportion of boys 32(94.12%) than girls 51(68.92%) reported eating mopane worms (p < 0.05). There were 310(62.50%) harvesters and significantly more adults 262(67.53%) than children 48(44.44%) were mopane worm harvesters (p < 0.05). Participants were required to select one main harvesting activity and picking of mopane worm on trees and on the ground 196(63.23%) was reported to be the most common while degutting of mopane worm was the least common activity 43(13.87%). While the majority of participants 206(66.67%) reported that they were involved in the harvesting of mopane worm at least 4 days a week, there were significant age and gender differences. The majority of children 30(62.5%) harvested no more than 3 days per week while more women than men harvested at least 4 days per week (p = 0.058). Only 51(16.45%) of the total study population reported the use of some form of PPE during harvesting. The most frequently reported forms of PPE were gloves and overalls. Self-reported mopane worm exposure factors were also stratified by cluster (Additional file 1: Table S2). Whilst mopane worm harvesting and post harvesting practices were not different among the 3 clusters, it was interesting to note that there was almost no mopane worm sensitisation (2%) in cluster 2 that consisted of *Tyrophagus putrescentiae* and *Alternaria alternata* sensitised individuals.

The results of the adjusted logistic regression analyses of the relationship between mopane worm sensitisation and self-reported exposure variables are presented in Table 4. Despite high p values, the direction of effect for most of the exposure variables was in line with our hypothesis. A mopane worm harvester was almost twice as likely to be sensitised to mopane worm (OR = 1.92, 95%CI = 0.77–4.79). Cooking or roasting during harvesting increased the odds of sensitisation (OR = 2.69, 95%CI = 0.78–9.31) when compared to a non-harvester. Harvesters not using PPE during harvesting were twice as likely to be sensitised compared to non-harvesters (OR = 2.12, 95%CI = 0.83–5.44) but the likelihood of sensitisation to mopane worm was reduced compared to non-harvesters among harvesters who reported the use of some form of PPE (OR = 1.18, 95%CI = 0.28–5.02). Harvesting for 2 or more years increased the likelihood of sensitisation to mopane worm compared to those that reported harvesting for less than 2 years (OR = 6.43, 95%CI = 0.83–49.85).

**Table 4:** Adjusted logistic regression models of factors associated with allergen sensitization

Exposure variables	Mopane worm sensitisation Adjusted OR(95%CI)^a^
Eat mopane worm	1.15 (0.48–2.79)
Harvest mopane worm	1.92 (0.77–4.79)
Both eat and harvest mopane worm	1.88 (0.61–5.84)
Specific harvesting activities	
Picking	1.83 (0.68–4.89)
Degutting	1.21 (0.21–7.01)
Cooking or roasting	2.69 (0.78–9.31)
PPE use during harvesting	
No PPE used	2.12 (0.83–5.44)
PPE used	1.18 (0.28–5.02)
Frequency of harvesting	
Up to 3 days/week	0.88 (0.26–3.03)
4–7 days/week	2.05 (0.80–5.29)
Harvesting for 2 or more years	6.43 (0.83–49.85)

Adjusted logistic regression models of factors associated with allergen sensitization

^a^Each OR(95%CI) represents a separate logistic regression model adjusted for age, gender, education, polysensitisation and family history of allergy

## Discussion

Our study is the first one reporting on population wide sensitization to mopane worm in a rural community in Zimbabwe. While there were more adults (78%) than children (22%) in the study population, the proportion of males compared to females was similar in both age groups. There was a higher response rate for skin prick testing (93.15%) than in the feasibility study (37%) [39]. We believe the response rate was increased by the continuous engagement with the community after completing the feasibility study as well as the use of the volunteer sampling method.

Sensitisation was predominantly to mopane worm (14.29%) and *Tyrophagus putrescentiae* (14.29%), closely followed by mopane leaves (13.42%) and both *Alternaria alternata* (6.49%) and *Dermatophagoides pteronyssinus* (6.49%). Sensitivity to *Tyrophagus putrescentiae* was almost exclusively in adults (17.22%) compared to children (3.92%). A high prevalence of *Tyrophagus putrescentiae* (72%) contrasted to mopane worm (50%) sensitisation was observed in the previously conducted feasibility study in Gwanda district [39]. *Tyrophagus putrescentiae* sensitization has been reported in Europe and Asia, and its clinical relevance, particularly in elderly subjects, is increasingly recognised [49]. Although co-sensitisation to *Dermatophagoides pteronyssinus* and/or *Dermatophagoides farina* is common among patients with sensitivity to *Tyrophagus putrescentiae* [50, 51], we are unaware of any studies addressing the molecular composition of mopane worms versus *Tyrophagus putrescentiae.* We speculate cross-reactive recognition of the two allergen sources as an explanation for the consistently high levels of *T. putrescentiae* reactivity in contrast with reactivity to *D. pteronyssinus* and *D. farinae.* Mite allergy due to *D. pteronyssinus* and *D. farinae* is common in Zimbabwe and has been associated with respiratory allergy [52]. While these data informed the inclusion of these allergens in a screening panel in Gwanda district in order to improve the detection rate of atopic individuals in the area, the dominance of *Tyrophagus putrescentiae* was unexpected and warrants further investigation.

Atopy and polysensitisation, which occur as a result of either cross reactivity or co-sensitisation [53], were more frequently observed in adults than children, particularly among women. Among the children, girls had higher sensitisation rates than boys. The proportion of self-reported allergy symptoms upon exposure to plants or furry animals and family history of allergy was generally higher in adults than in children. The cross sectional nature of the study design and the use of non-probability sampling, however, limits us from concluding that there is increasing sensitisation with increase in age. It is well documented, though, from longitudinal studies that the prevalence of allergic sensitization increases with increasing age from childhood to early adulthood and then starts to decrease thereafter [54]. The high level of polysensitisation among adults, especially women, was a cause for concern because of the associated increase in risk and severity of allergic diseases [53]. Allergy can develop at any point in life, including in adulthood, due to exposure to environmental, occupational and lifestyle factors [55]. Furthermore, dependence on the abundant and diverse local flora and fauna for food and other purposes in African communities influences the observed allergy profile [56]. It was not surprising that women, who are often responsible for several activities including mopane worm harvesting to ensure their families’ wellbeing, were the most sensitised subgroup in this study population.

Cluster analysis was used to identify the natural grouping of allergens amongst the participants. It was found that mopane worm, mopane leaves and *Tyrophagus putrescentiae* tended to cluster together in adults. While there was frequent co-sensitisation between mopane worms and mopane leaves, probably due to co-exposure, there were also some cases of monosensitivity to both extracts. The frequent co-sensitisation to mopane worm and *Tyrophagus putrescentiae* extracts could be due to the existence of cross reactive molecules or reactivity to pan-allergens such as tropomyosin [57]. Such cross-reactivity is likely to involve allergens that are not represented in both *D. pteronyssinus* and D*. farinae* that were also tested in this population. Additionally, we also believe there could also be a case of co-exposure between the allergens*. Tyrophagus putrescentiae*, a type of storage mite, is a pest of many high fat or protein content foods [58] and could possibly contaminate mopane worm during storage [59, 60].

Mopane worm harvesting and post harvesting practices were similar among the 3 clusters and yet there was almost no mopane worm sensitisation (2%) in cluster 2 that consisted mostly of *Tyrophagus putrescentiae* and *Alternaria alternata* sensitised adults. It has been reported that pre-existing IgE reactivity profile does not seem to change substantially in adulthood and the limited neo-sensitisation to mopane worm that was observed in this cluster supports the phenomenon [61]. Prevalence of mopane worm sensitisation, however, was high amongst adults in cluster 3 (96.88%) relative to cluster 2. The longer duration of exposure through harvesting and post-harvesting activities that was reported in cluster 3 could be the influencing variable. While cluster analysis was able to provide insight into underlying co-sensitisation patterns, there are some methodological limitations to consider. Cluster analysis is exploratory in nature and there are several different clustering algorithms to choose from. We selected Ward’s method of hierarchical clustering as it has been used in other studies assessing allergen clusters in different populations [62]. Agglomerative hierarchical clustering includes all observations into a cluster and even though three clusters were optimally selected, there are likely smaller, but important, sub clusters within the main clusters [63]. To establish whether a positive mopane worm skin prick test was due to true sensitization or cross-reactivity would require the use of component-resolved diagnosis (CRD), a more specific diagnosis which utilises pure allergenic molecules as opposed to a natural extract [64, 65].

There is substantial evidence from occupational studies that exposure to insect and insect-derived materials result in sensitization that subsequently leads to elicitation of respiratory allergy [33]. While studies on edible insects have focused on sensitisation by oral exposure [32], it would appear that dermal or inhalation exposure are also important [66] as a result of processing activities that need to take place before the insects are actually consumed [67–69]. The exposure scenario was useful in depicting the various opportunities of exposure to the mopane worms. The majority of participants (70.77%) reported that they eat mopane worm and 62.5% reported that they harvest mopane worm. An entomophagy survey in Zimbabwe concluded that consumption of mopane worm has remained undiminished over the years across the country [25]. Interestingly, while it was found in this study that girls had higher sensitisation rates than boys, there were significantly more boys than girls who reported eating and harvesting mopane worm. Our exposure assessment, however, was limited since it was based on self-reports and prone to recall bias. As a result, there were a number of missing values particularly for variables that needed a fair amount of recall such as the frequency of harvesting per week and the lifetime duration of harvesting. A more robust longitudinal study is required to further explore these findings.

The results from the adjusted logistic regression models between mopane worm sensitisation and selected exposure variables were in line with our hypothesis that extensive contact with mopane worm through various activities could lead to sensitisation. Mopane worm exposure through harvesting and post harvesting practices increased the likelihood of mopane worm sensitisation regardless of age, gender, education, polysensitisation and family history of allergy. Cooking or roasting during harvesting seemed particularly influential compared with other specific harvesting processes (OR = 2.69, 95%CI = 0.78–9.31). A possible explanation for this could be the resultant exposure to inhalable steam, aerosols or dust particles during the boiling and roasting of mopane worms [20, 27]. Exposure to food allergens by inhalation during preparation is believed to have contributed to the increasing prevalence of respiratory allergy in the food industry [66, 70]. Nevertheless, none of the reported adjusted odds ratios were statistically significant, suggesting insufficient evidence to reject the null hypothesis that there is no relationship between exposure and sensitisation to mopane worm. The use of non-probability sampling in the recruitment of participants could weaken the study, although this could not be avoided, it potentially limits the generalizability of the study findings. Notwithstanding this, we believe that our findings are a reasonable representation of sensitisation patterns in Gwanda district and other areas with similar environmental exposures. We also raise pertinent research questions with respect to cross-reactive recognition of other allergenic molecules including *Tyrophagus putrescentiae.*

## Conclusion

Our study provides useful insights about the underlying sensitisation patterns in the rural community of Gwanda district in Zimbabwe. We established that allergic sensitization was more common in adults than children, especially among women. Co-sensitivity to mopane worm and mopane tree leaves was more frequent particularly in children. Amongst adults, there was co-sensitivity between mopane worm, mopane tree leaves and *Tyrophagus putrescentiae*. If these allergens are included in the screening panel, they could improve the detection rate of atopic individuals in Gwanda district and other similar areas. Monosensitisation to mopane worm suggests that mopane worms could contain unique allergenic proteins whereas co-sensitisation with other extracts indicates co-exposure or cross-reactivity.

Two important research questions emerge from this study. Firstly, further investigation is required to identify and characterize the allergenic proteins in mopane worm. Secondly, the clinical relevance of mopane worm sensitisation and co-sensitisation should be assessed in order to inform interventions that promote safety and hygiene practices that limit health risks throughout the harvesting and post harvesting processes.

## Supplementary Information

**Additional file 1.** Summary of allergen cluster analysis and profiling. **Fig S1.** A dendrogram produced from agglomerative hierarchical cluster analysis using Ward’s method. **Table S1.** Demographic characteristics and sensitisation patterns by cluster. **Table S2.** Harvesting and post harvesting practices among the clusters.

## Data Availability

The datasets used and/or analysed during the current study are available from the corresponding author on reasonable request.

## Abbreviations

GARAS: Gwanda Asthma and Respiratory Allergy Study
CE: Community engagement
SPT: Skin prick test
IgE: Immunoglobulin E
PPE: Personal protective equipment
