# Supplementary material for: Sensitisation to Imbrasia belina (mopane worm) and other local allergens in rural Gwanda district of Zimbabwe
Source: Allergy Asthma Clin Immunol. 2022 Apr 9;18:33. doi: 10.1186/s13223-022-00668-0 (PMC8994392; doi:10.1186/s13223-022-00668-0)
Supplement: Supplementary file 1 — Additional file 1. Summary of allergen cluster analysis and profiling. Fig S1. A dendrogram produced from agglomerative hierarchical cluster analysis using Ward’s method. Table S1. Demographic characteristics and sensitisation patterns by cluster. Table S2. Harvesting and post harvesting practices among the clusters. [file 13223_2022_668_MOESM1_ESM.docx]

**Additional file 1: Cluster analysis and profiling**

**
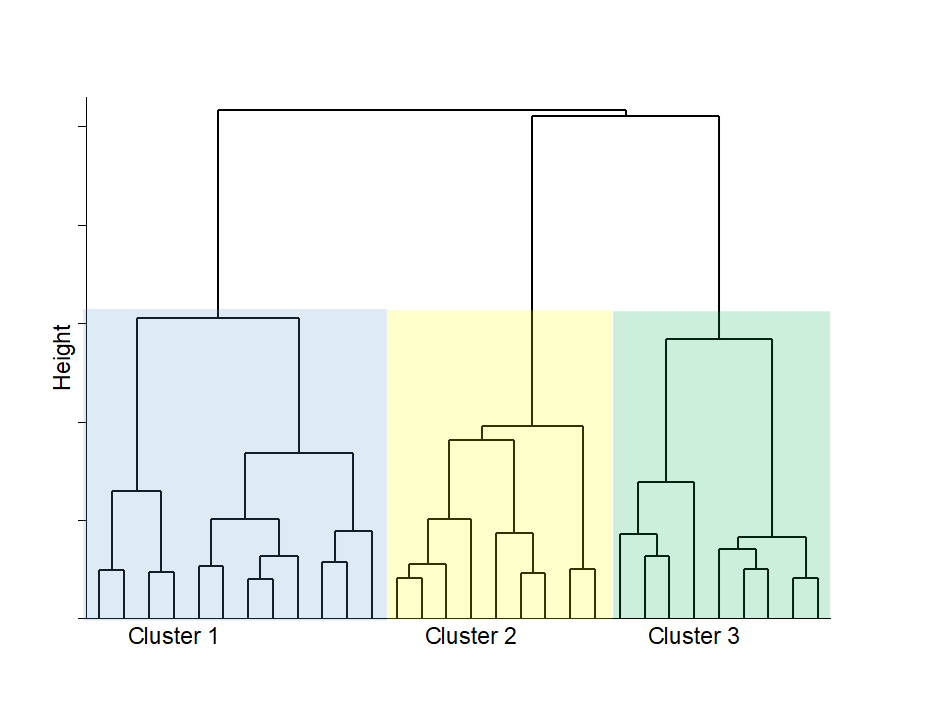
**Fig S1: A dendrogram produced from agglomerative hierarchical cluster analysis using Ward’s method. Height is a rep­resentation of similarity: the lower the number at the point of conver­gence, the more similar the clusters. Using the Caliński and Harabasz pseudo-F index and a visual assessment of the dendogram, allergens were optimally divided into three clusters

In Table S1, we summarise the characteristics of each cluster. The boldface values highlight the most abundant allergens in each cluster.

In summary, we find that:

- Cluster 1: Dominant allergens are mopane worm and mopane leaves
- Cluster 2: Dominant allergens are *Tyrophagus putrescentiae* and *Alternaria alternata*
- Cluster 3: Dominant allergens are mopane worm, mopane leaves, *Tyrophagus putrescentiae* and Trees mixture.

**Table S1: Demographic characteristics and sensitisation patterns by cluster**

|  | Cluster 1 (n=63), n (%) | Cluster 2 (n=49), n (%) | Cluster 3 (n=32), n (%) | Total (n=144), n (%) |
| --- | --- | --- | --- | --- |
| Adults, n (%)* | 46(73.02) | 45(91.84) | 29(90.63) | 120(83.33) |
| Adults age (years), median(IQR) | 41(29-53) | 47(33-66) | 49(36-64) | 44(33.5-60) |
| Children, n (%) | 17(26.98) | 4(8.16) | 3(9.38) | 24(16.67) |
| Children age (years), median(IQR) | 14(13-16) | 14.5(12-15.5) | 17(11-17) | 14.5(12.5-16) |
| Gender, n (%)* |  |  |  |  |
| Female | 52(82.54) | 31(63.27) | 20(62.50) | 103(71.53) |
| Male | 11(17.46) | 18(36.73) | 12(37.50) | 41(28.47) |
| Polysensitisation, median(IQR)** | 2(1-3) | 2(1-3) | 4(4-7) | 2(1.5-4) |
| **Sensitisation to specific allergens, n (%)** | | | | |
| Barley** | 5(7.94) | 9(18.37) | 12(37.5) | 26(18.06) |
| 5grassmixture** | 0(0) | 3(6.12) | 10(31.25) | 13(9.03) |
| Treesmixture** | 7(11.11) | 6(12.24) | **13(40.63)** | 26(18.06) |
| Cockroach** | 16(25.40) | 0(0) | 9(28.13) | 25(17.36) |
| Mosquito | 14(22.22) | 3(6.12) | 5(15.63) | 22(15.28) |
| HDMfar | 9(14.29) | 5(10.20) | 9(28.13) | 23(15.97) |
| HDMpter** | 6(9.52) | 14(28.57) | 10(31.25) | 30(20.83) |
| Tyrophagus** | 3(4.76) | **35(71.43)** | **28(87.50)** | 66(45.83) |
| Alternaria** | 0(0) | **18(36.73)** | 12(37.50) | 30(20.83) |
| Weedmixture** | 4(6.35) | 6(12.24) | 9(28.13) | 19(13.19) |
| Mopane worms** | **34(53.97)** | 1(2.04) | **31(96.88)** | 66(45.83) |
| Mopane leaves** | **32(50.79)** | 7(14.29) | **23(71.88)** | 62(43.06) |
| *p<0.05  **p<0.001 | | | | |

Additionally, participants in Cluster 3 were older and had significantly higher levels of polysensitisation than in the other clusters. Most children belonged to cluster 1.

**Table S2: Harvesting and post harvesting practices among the clusters**

|  | Cluster 1 (n=63), n (%) | Cluster 2 (n=49), n (%) | Cluster 3 (n=32), n (%) | Total (n=144), n (%) |
| --- | --- | --- | --- | --- |
| Eat mopane worm | 40(63.49) | 31(63.27) | 21(65.63) | 92(63.89) |
| Harvest mopane worm* | 38(60.32) | 33(67.35) | 21(65.63) | 92(63.89) |
| Picking mopane worm | 23(36.51) | 23(46.94) | 14(43.75) | 60(41.67) |
| Degutting mopane worm | 6(9.52) | 3(6.12) | 1(3.13) | 10(6.94) |
| Cooking mopane worm | 9(14.29) | 7(14.29) | 6(18.75) | 22(15.28) |
| PPE during harvesting | 5(7.94) | 7(14.29) | 5(15.63) | 17(11.81) |
| No PPE during harvesting | 33(52.38) | 26(53.06) | 16(50) | 75(52.08) |
| Harvesting/week* |  |  |  |  |
| 1 day | 1(2.63) | 1(3.03) | 1(4.76) | 3(3.26) |
| 2-3 days | 10(26.32) | 10(30.30) | 2(9.52) | 22(23.91) |
| 4-7 days | 27(71.05) | 22(66.67) | 18(85.71) | 67(72.83) |
| Years of harvesting, median(IQR) | 5(3-15) | 10(3-15) | 14(5-15) | 8(3-15) |
| *p<0.05 | | | | |

In Table S2, a comparison of mopane worm harvesting and post harvesting activities across the 3 clusters indicates that the practices were similar and yet there was limited sensitisation to mopane worm in cluster 2.
